# Inhibition of Bone Resorption and Anti-Inflammatory Effect of 7-Hydroxycoumarin in an Animal Model of Lipopolysaccharide-Induced Periodontal Disease

**DOI:** 10.1155/ijod/9971146

**Published:** 2025-07-24

**Authors:** Luiza Teles Barbalho Ferreira, Renan Fernandes do Espírito-Santo, Milena Botelho Pereira Soares, José Maria Barbosa-Filho, Cristiane Flora Villarreal, Paulo José Lima Juiz

**Affiliations:** ^1^Federal University of Recôncavo of Bahia, Feira de Santana, Bahia, Brazil; ^2^School of Dentistry, University of Maryland Baltimore, Baltimore, Maryland, USA; ^3^Gonçalo Moniz Research Center, Salvador, Bahia, Brazil; ^4^Federal University of Paraíba, João Pessoa, Paraíba, Brazil; ^5^Faculty of Pharmacy, Federal University of Bahia, Salvador, Bahia, Brazil

**Keywords:** immune response, periodontitis, umbelliferone

## Abstract

**Introduction:** Periodontal disease (PD) has a multifaceted etiology. The immune response to microbial invasion triggers osteoclast activation, leading to the resorption of alveolar bone and ultimately resulting in tooth loss.

**Objective:** This study aims to investigate the anti-inflammatory properties of the bioactive compound 7-hydroxycoumarin (7HC) at concentrations of 50 and 100 mg/kg. We seek to evaluate its effectiveness in controlling alveolar bone resorption in treating PD induced by lipopolysaccharide (LPS) in an animal model.

**Methodology:** PD was induced in C57 Bl/6 mice using a LPS injection protocol. The mice were randomly assigned to different treatment groups, receiving 7HC at 50 and 100 mg/kg, nimesulide at 25 mg/kg, and a control group that received the vehicle. Treatments were administered once daily from the 14th to the 28th day of the study. Following this treatment period, the animals were euthanized, and gingival tissue samples were collected to assess cytokine production (interleukin-1β [IL-1β] and interleukin-6 [IL-6]) using enzyme-linked immunosorbent assay (ELISA). Additionally, mRNA expression levels of matrix metalloproteinase-9 (MMP-9), TIMP-1, IL-10, and TGF-β were analyzed through qRT-PCR. The maxillae were extracted for the quantification of alveolar bone resorption by measuring the most significant distance between the cementoenamel junction and the alveolar bone crest (ABC) utilizing ImageJ software.

**Results:** The bioactive 7HC effectively inhibited the expression of IL-1β, IL-6, and MMP-9, while simultaneously increasing the mRNA expression of IL-10 and TGF-β, cytokines recognized for their anti-inflammatory properties. Histometric analysis of the alveolar bone indicated that 7HC protects alveolar bone resorption in an animal model of PD induced by LPS injection.

**Conclusion:** The bioactive 7HC not only inhibited proinflammatory cytokines but also stimulated the expression of the anti-inflammatory cytokine, thereby preventing bone resorption. This positions 7HC as a promising candidate for periodontal therapy.

## 1. Introduction

Periodontal diseases (PDs) include a range of inflammatory conditions that affect the protective and supportive structures of the teeth, potentially leading to tooth loss over time [1]. This chronic condition is multifactorial, arising from an immune response to a dysbiotic biofilm in the subgingival area [2]. Both genetic predisposition and environmental factors can significantly influence the progression of periodontitis [3]. The immune response directed at periodontopathogens leads to the activation of osteoclasts, resulting in the pathological resorption of alveolar bone and the subsequent loss of dental elements [4, 5]. This emphasizes the importance of controlling inflammatory processes; thus bioactive compounds extracted from medicinal plants are a viable alternative.

Many studies have established a significant link between periodontitis and overall systemic health in recent decades. This connection indicates that periodontitis can serve as a risk factor for the development or exacerbation of various conditions, including diabetes and cardiovascular diseases [6], complications during pregnancy [7], ischemic strokes [8], and Alzheimer's disease [9] among others. Consequently, PD is recognized as a significant public health issue.

Antiseptics, particularly those based on chlorhexidine and systemic medications to address infection and inflammation, have become common adjuncts to scaling, root planing, and/or surgical interventions for PD [10]. However, although chlorhexidine-based mouthwash is effective, it is associated with several side effects, such as xerostomia, hypogeusia, and a discoloration of tongue; as well as calculus and extrinsic tooth staining in long-term use [10, 11], and disruption of the oral microbiome [12]. Additionally, existing literature points to antibiotic resistance among pathogens linked to the disease [13], underscoring the necessity for alternative therapies or adjuncts to conventional treatments [14]. In this context, medicinal plants and their bioactive chemical constituents may offer a promising alternative.

Exploring medicinal plants as an adjunctive treatment to scaling and root planing therapy has gained significant attention in dentistry. A study conducted by Gościniak et al. [15] highlights that the incorporation of bioactive compounds derived from medicinal plants alongside conventional therapy can enhance treatment efficacy while mitigating the adverse effects commonly associated with traditional agents like chlorhexidine. Furthermore, they discuss various products already available on the market, including toothpaste, antiseptics, and oral sprays, illustrating that phytotherapy is not just a theoretical concept but a practical reality in dental care.

In this context, coumarins, found abundantly in medicinal plants, show significant pharmacological potential. These compounds have antioxidant [16, 17], anti-inflammatory [18], and antimicrobial properties [19]. Given the role of inflammation in PD, using natural products like coumarins with anti-inflammatory properties could offer a promising treatment strategy.

Among coumarins, auraptene and lacinartin are polyphenols in the coumarin family. Auraptene has been shown to significantly reduce the secretion of IL-8 and TNF-α in LPS-stimulated macrophages. Similarly, at a 50 μg/mL concentration, lacinartin also effectively inhibits the secretion of IL-8 and TNF-α. Furthermore, both auraptene and lacinartin contribute to a reduction in the secretion of matrix metalloproteinase-8 (MMP-8) and matrix metalloproteinase-9 (MMP-9) [20].

Umbelliferone (7-hydroxycoumarin [7HC]), a derivative of coumarin, also demonstrates the ability to attenuate the secretion of proinflammatory cytokines and chemokines in HaCAT cells treated with TNF-α and IFN-γ. This attenuation is mediated through the inhibition of IκBα degradation, the nuclear translocation of NF-κB, and the phosphorylation of STAT1, indicating a dose-dependent response to umbelliferone treatment [21].

Additionally, intravenously injected gelatin-coated ZnO–ZnS core–shell nanoparticles containing 7HC, reduced the production of proinflammatory cytokines, such as interleukin-1β (IL-1β), interleukin-6 (IL-6), and IL-17, along with prostaglandin E (PEG2) [22].

It is important to highlight that oxidative stress significantly worsens inflammation and accelerates the progression of periodontitis. Umbelliferone (7HC) can effectively scavenge free radicals and inhibit lipid peroxidation [23, 24]. The antioxidant properties of 7HC are likely the result of activating the Nrf2 signaling pathway, which enhances the activities of endogenous antioxidants, such as superoxide dismutase, glutathione, catalase, and NAD (P)H-quinone oxidoreductase [23, 25, 26].

In addition to its anti-inflammatory and antioxidant benefits, 7HC has demonstrated the ability to inhibit the formation of biofilms produced by uropathogenic *Escherichia coli* at a concentration of 50 μg/mL. It also effectively reduced biofilm formation by methicillin-resistant strains of *Staphylococcus epidermidis* at a concentration of 500 μg/mL. Considering the significant role that dysbiotic biofilms play in the development of PD, the antimicrobial properties of 7HC show great promise for managing this condition [24, 27].

This study aims to evaluate the anti-inflammatory effects of 7HC and its ability to prevent alveolar bone resorption in an animal lipopolysaccharide (LPS)-induced PD model.

## 2. Materials and Methods

### 2.1. Ethical Aspects

The animal testing conducted in this study received approval from the Ethics Committee on the Use of Animals (CEUA) at FIOCRUZ, in accordance with the resolution established by the National Council for the Control of Animal Experimentation (CONCEA) regarding the responsible use of animals (CONCEA, 2015). This approval is documented under registration number 012/2022.

### 2.2. Experimental Section

The 7HC active compound collected, extracted, and isolated from *T. domingensis* were previously detailed [28], and used in the present study.

To assess the inhibition of bone resorption and the anti-inflammatory properties of 7HC, PD was induced in C57Bl/6 mice sourced from the CPqGM Animal Facility at Fiocruz Bahia. This procedure was modified from the protocol used for inducing PD as described in [29].

The mice had a weight range of 25–30 g and were between 8 and 10 weeks old. They were kept in a temperature-controlled environment (22–25°C) under a 12-h light/dark cycle, with unrestricted access to food and water throughout the experimental duration.

The mice were randomly assigned to five distinct groups, with each group consisting of 12 animals: Group 1: Naïve group—animals that received no treatment and had no induced PD; Group 2: Negative control group—animals treated with a vehicle (saline + 5% DMSO) and subjected to induced PD; Group 3: Animals treated with 7HC at 50 mg/kg and induced PD; Group 4: Animals treated with 7HC at a 100 mg/kg dosage and induced PD; Group 5: Positive control group—animals treated with nimesulide at 25 mg/kg and with induced PD.

Mice were anesthetized with an intraperitoneal injection of ketamine (5 mg/kg) and xylazine (20 mg/kg). Once adequate anesthesia was achieved, each mouse received 1 µL of 20 µg/µL of LPS derived from *Escherichia coli*, diluted in injection water.

This injection was administered into the gingival papilla located between the first and second upper left molars. The procedure was carried out on alternate days over a 21-day period as part of a protocol designed to induce PD [29].

The methodology, adapted from Trombetta-Silva et al. [29], recommends a 4-week injection regimen of 2 µL of LPS (10 µg/µL) isolated from *Aggregatibacter actinomycetemcomitans*, diluted in phosphate-buffered saline (PBS). In contrast, this study employed 1 µL of LPS (20 µg/µL) isolated from *Escherichia coli*, diluted in injection water, over the 21-day duration.

A powdered form of 7HC, diluted in a saline solution with 5% DMSO, was utilized to prepare the dosages of 7HC. Beginning 14 days after the onset of disease induction, the mice were treated orally via gavage with 7HC at dosages of 50 and 100 mg/kg, or with its vehicle (5% DMSO diluted in saline). Nimesulide (Sigma–Aldrich), a nonsteroidal anti-inflammatory drug (NSAID) commonly used in dentistry to manage inflammatory conditions, served as the gold standard drug in this study [30].

The treatment was administered once daily until the experiment concluded at 28 days. Following the treatment period, the animals in each group were euthanized using a combination of xylazine hydrochloride (1 mg/kg) and ketamine (15 mg/kg), followed by an anesthetic overdose of sodium thiopental (50 mg/kg) and intravenous administration of potassium chloride (2.56 mEq/kg).

The excised gingival tissue samples were preserved in 1 mL of trizol and promptly utilized for reverse transcription polymerase chain reaction (RT-PCR) assays to ensure the integrity of the RNA. In contrast, for the enzyme-linked immunosorbent assay (ELISA) protocols, the samples were stored in a buffer solution at −80°C to maintain their viability and prevent degradation.

### 2.3. ELISA

Gingival tissue samples were homogenized in PBS at 100 mg of tissue per mL. Subsequently, the following components were added: 0.4 M NaCl, 0.05% Tween 20, and protease inhibitors, which included 0.1 mM PMSF, 0.1 mM benzethonium chloride, 10 mM EDTA, and 20 KI of aprotinin A per 100 mL (Sigma). The samples were centrifuged for 10 min at 3000 *g*, and aliquots of the supernatant were frozen at −80°C for future quantification.

The cytokines IL-1β, IL-6, and IL-10 were quantified using the sandwich ELISA technique with Duoset ELISA Development System kits from R&D Systems (Minneapolis, USA) for each cytokine [31]. Ninety-six-well plates (NUNC – IMMUNO PLATE Maxisorp Surface) were sensitized by adding 50 μL of capture antibody (a purified monoclonal antibody specific to the investigated cytokine), diluted in PBS, and incubated at 4°C overnight. The plates were washed three times with PBS containing 0.05% Tween 20. Next, 100 μL of a 1% PBS-BSA solution were added to each well and incubated for 2 h at room temperature to block non-specific binding sites. After one additional wash, the plates were incubated with 50 μL of each sample (in duplicate) and standard cytokines at various concentrations for 12 h at 4°C.

After washing the plates, biotinylated anticytokine antibodies were introduced, and the plates were incubated for an additional 2 h at room temperature. Following this incubation, the plates were rewashed, and 50 μL of streptavidin, diluted 1:200, were added for 20 min at room temperature. After another wash, the reaction was developed using 50 μL of a revealing solution composed of 10 mL of 1M citrate-phosphate buffer, 2 μL of H_2_O_2_, and one TMB (tetramethylbenzidine) tablet. The reaction was subsequently blocked by adding 50 μL of a 1:20 phosphoric acid solution. Optical density was measured using a spectrophotometer (Spectra Max 190—Molecular Devices, California, USA) at a wavelength of 450 nm. The analysis was conducted using Softmax 4.3.1 Software (Molecular Devices).

### 2.4. Real-Time PCR

The mRNA expression levels of IL-1β, IL-6, MMP-9, TIMP-1, IL-10, and TGF-β in gingival tissue were assessed using real-time polymerase chain reaction (qRT-PCR). RNA was extracted using TRIzol/chloroform reagents (Invitrogen, Carlsbad, CA, USA), and its concentration was determined with the Nanodrop 2000 apparatus. According to the manufacturer's instructions, cDNA was synthesized from 1 μg of RNA using the High Capacity cDNA Reverse Transcription Kit (Applied Biosystems, Foster City, CA, USA) [32].

For the gene expression assay, the following TaqMan probes were utilized: il1b (Mm_0043228_m1), 6 (Mm_00446190_m1), il10 (Mm_00439616_m1), mmp9 (Mm_00442991_m1), tgfβ (Mm_01178820_m1), and timp1 (Mm_01341361_m1), along with negative controls. All reactions were conducted in duplicate and analyzed using the ABI7500 Sequence Detection System (Applied Biosystems) under standardized thermal amplification conditions specified by the manufacturer.

The mean cycle threshold (Ct) value obtained from duplicate measurements was used to calculate the expression level of the target gene. It was done by normalizing with the endogenous gene Gapdh (mm99999915_g1) using the formula 2^ (-*Δ*Ct). Any Cts with a coefficient of variation greater than 5% were excluded from the analysis.

### 2.5. Analysis of Alveolar Bone Resorption

Morphometric analyses were performed to assess alveolar bone resorption associated with the experimental disease. The excised maxillae were fixed in 10% formalin for 24 h. Subsequently, they were divided into two hemiarches (right and left), meticulously dissected, and stained with 1% methylene blue to differentiate the bone tissue from the teeth, as the latter stains less intensely.

Photographs of the lingual surface of the stained first molars was taken using a Leica camera paired with a stereomicroscope to evaluate the extent of alveolar bone loss following disease induction. The greatest distance between the cementoenamel junction and the alveolar bone crest (ABC) was measured utilizing ImageJ software (National Institutes of Health, USA) [33].

### 2.6. Statistical Analyses

The results are expressed as the mean ± standard deviation (SD) based on analyses conducted with groups of animals. The normality of the data was evaluated using the Kolmogorov–Smirnov test. For data that exhibited a normal distribution, comparisons between groups were performed using a one-way ANOVA with Tukey's post hoc test. Conversely, for data that did not conform to normality, comparisons were made utilizing the nonparametric Friedman test, followed by pairwise post hoc comparisons using the pairwise method with Bonferroni correction.

The ELISA and RT-PCR results were analyzed using Prism 7 software (GraphPad, San Diego, CA, USA). At the same time, alveolar bone loss was evaluated using IBM SPSS Statistics software. A *p*-value of less than 0.05 was considered statistically significant.

## 3. Results and Discussion

The results of this study indicate that 7HC has anti-inflammatory activity, offering protection against alveolar bone resorption in an animal model of LPS-induced PD.

To evaluate the effectiveness of inflammation induction via LPS injection, proinflammatory cytokine levels of IL-1β and IL-6 were measured using ELISA. The results demonstrated an increase in the expression of both cytokines in animals with induced PD treated with the vehicle compared to those in the naïve group. This indicates that the animal model employed in the study successfully stimulated active inflammation.

**Figure 1:**
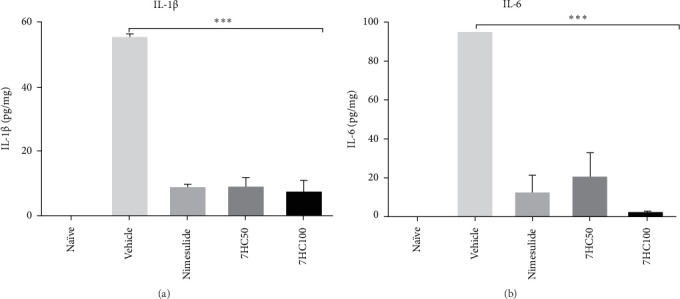
Impact of 7-hydroxycoumarin (7HC) treatment at concentrations of 50 and 100 mg/kg on the expression of proinflammatory (A, B) cytokines in gingival tissue excised from C57BL/6 mice with experimental periodontal disease. Data are presented as mean ± standard deviation. Statistical significance is indicated at *p*  < 0.0001^*∗∗∗*^.

The inflammatory response was controlled by administering the standard drug (nimesulide at 25 mg/kg) and 7HC at 50 and 100 mg/kg (*p*  < 0.0001). Notably, no statistically significant differences existed between the groups treated with these compounds (Figure 1).

In this study, LPS, a key component of the outer membrane of Gram-negative bacteria, was used to induce periodontitis. Toll-like receptors (TLRs) identify LPS and initiate the inflammatory response. The TLR-CD14-LPS-MyD88 activation cascade is vital to the innate immune response, particularly in identifying bacterial pathogens. LPS is initially detected by the CD14 receptor, which is present on the surface of immune cells like macrophages and dendritic cells. This interaction enhances the binding of LPS to TLR4, initiating intracellular signaling. Following this activation, the MyD88 protein is recruited to the TIR (Toll/IL-1 receptor) domain of TLR4, which facilitates the assembly of a signaling complex that includes additional adaptor proteins, such as IRAK (interleukin-1 receptor-associated kinase) and TRAF (TNF receptor-associated factor). Signaling through MyD88 activates a pathway that ultimately leads to the activation of the transcription factor NF-kB, promoting the transcription of genes that produce inflammatory cytokines, including IL-1β and IL-6, amplifying the inflammatory response. While this cascade is crucial for the body's defense, it can also damage tissue if not adequately regulated [34, 35].

IL-1β is a key proinflammatory cytokine secreted mainly through monocytes/macrophages and dendritic cells in response to pathogen-associated molecular patterns (PAMPs), such as LPS [34]. IL-6 is a proinflammatory cytokine and its concentration in gingival crevicular fluid was elevated in individuals diagnosed with chronic periodontitis compared to those who are periodontally healthy. This suggests a potential link between increased IL-6 levels and the inflammatory processes associated with chronic periodontitis, highlighting the role of this cytokine as a marker of PD progression [36, 37].

A cohort study [38] comprising 68 patients diagnosed with PD, carried out from 2021 to 2022, uncovered a positive correlation between heightened levels of the proinflammatory cytokines IL-1β and IL-6 in the patients' saliva and severe clinical parameters. Specifically, the study found that these elevated cytokine levels were associated with increased probing depth (PPD), indicating the severity of periodontal tissue destruction and a higher plaque index (PI). Additionally, increased bleeding on probing (BOP) was observed, signaling inflammation in the gum tissue. Furthermore, the study revealed a reduction in the clinical attachment level (CAL), highlighting the loss of supportive periodontal tissue and attachment to the teeth.

**Figure 2:**
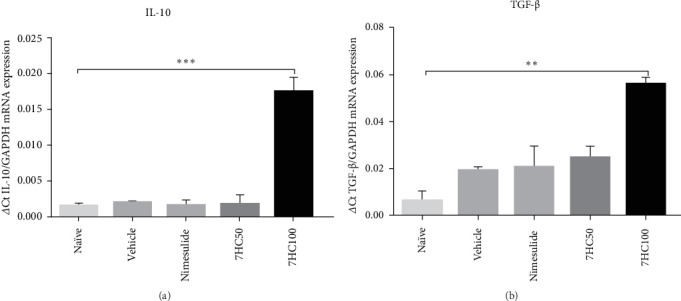
Impact of 7-hydroxycoumarin (7HC) treatment at concentrations of 50 and 100 mg/kg on the mRNA expression of anti-inflammatory (A, B) cytokines in gingival tissue excised from C57BL/6 mice with experimental periodontal disease. Data are presented as mean ± standard deviation. Statistical significance is indicated at *p*  < 0.001^*∗∗*^; *p*  < 0.0001^*∗∗∗*^.

Treatment with 7HC at 50 and 100 mg/kg reduced the expression of proinflammatory cytokines IL-1β and IL-6, while increasing the expression of mRNA for anti-inflammatory cytokines. This study revealed that the inflammatory stimulus induced by LPS in animals with PD, treated with a vehicle, suppressed mRNA expression for the anti-inflammatory cytokines IL-10 and TGF-β. Remarkably, the standard drug nimesulide at a dosage of 25 mg/kg failed to reverse this suppression. In contrast, treatment with 7HC at 100 mg/kg effectively induced mRNA expression for IL-10 (*p*  < 0.0001) and TGF-β (*p*  < 0.001) compared to both the vehicle and nimesulide groups. This finding underscores the protective effect of 7HC in the animal model of PD induced by LPS (Figure 2).

IL-10 is crucial for regulating the inflammatory response in PD, as it reduces inflammation and helps to prevent excessive tissue damage [39]. Meanwhile, TGF-β promotes tissue healing in areas affected by PD by stimulating the production of extracellular matrix components, such as collagen. It also enhances the synthesis of proteinase inhibitors and inhibits the production of collagenases [40].

TGF-β facilitates the migration and differentiation of osteoblast precursors [41], thereby stimulating the production of bone matrix. Moreover, it induces B lymphocytes to differentiate into plasma cells that produce IgA [42]. IgA is essential for protecting mucous membranes, which is particularly pertinent in periodontal therapy.

Studies have documented the effects of coumarins on osteoclasts. For instance, urolithin *b* suppresses osteoclast activation and reduces bone loss of osteoporosis [43]. Furthermore, the in vitro effects of *Cnidium monnieri* extract, comprised 80% coumarins, successfully inhibited the differentiation of osteoclast precursors into active osteoclasts [44].

**Figure 3:**
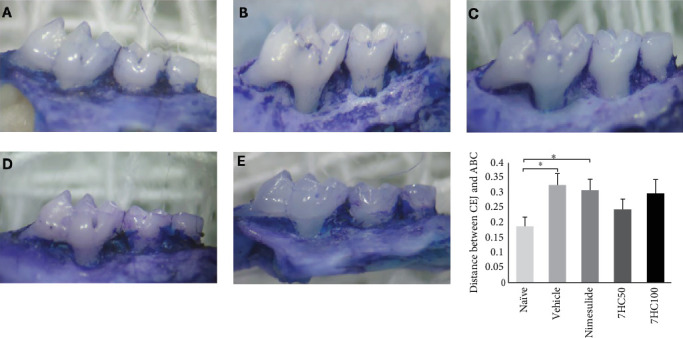
The impact of the compound 7-hydroxycoumarin (7HC) on alveolar bone resorption in the maxilla of C57BL/6 mice with induced periodontal disease. Panels (A-E) showcase representative images from various treatment groups: (A) naïve, (B) vehicle, (C) nimesulide, (D) 7HC at 50 mg/kg, and (E) 7HC at 100 mg/kg. The graph compares bone resorption caused by lipopolysaccharide (LPS), with that treated by 7-hydroxycoumarin (7HC). The following key terms are defined: ABC, alveolar bone crest; CEJ, cementoenamel junction. Data are presented as mean ± standard deviation, with statistical significance denoted as *⁣*^*∗*^*p*  < 0.05.

In line with this idea, to assess whether 7HC effectively controlled the progression of bone resorption, the distance between the cementoenamel junction (CEJ) and the ABC was measured. The results demonstrated that the distance between the CEJ and ABC in animals treated with the vehicle was significantly greater than that observed in the naïve group (*p*  < 0.05). Although treatment with 7HC at 50 and 100 mg/kg did not stop bone resorption progression, it suggests a protective effect. This conclusion is reinforced by the fact that the distance between the CEJ and ABC in the treated animals did not differ statistically from that of the naïve group (Figure 3).

The protective effect against bone resorption can be attributed to the anti-inflammatory properties of 7HC. Specifically, interleukin-6 can stimulate the expression of receptor activator of nuclear factor-kappa *B* ligand (RANKL) in osteoblasts and osteocytes [45]. RANKL is a crucial signaling molecule that belongs to the tumor necrosis factor superfamily and is secreted by various cell types, including osteoblasts, bone marrow stromal cells [46], and lymphocytes [47]. Once released, RANKL binds to the RANK receptor located on the surface of osteoclast precursors. This interaction is important for promoting the differentiation of these precursors into mature osteoclasts, the specialized cells responsible for bone resorption [48]. Thus, IL-6 also contributes to inflammatory processes and plays a pivotal role in bone remodeling through its influence on RANKL and osteoclast biology.

The alveolar bone resorption process is influenced by MMPs-9, an enzyme that serves as an important indicator of PD progression. MMP-9 degrades collagen types IV, V, and XI, along with proteoglycans and elastin, thus playing a critical role in tissue destruction [49].

**Figure 4:**
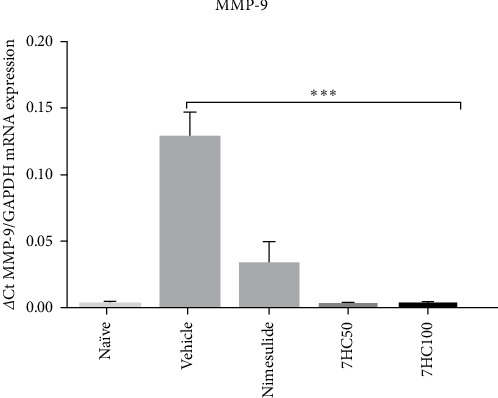
Impact of 7-hydroxycoumarin (7HC) treatment at concentrations of 50 and 100 mg/kg on the mRNA expression of matrix metalloproteinases-9 in gingival tissue excised from C57BL/6 mice with experimental periodontal disease. Data are presented as mean ± standard deviation. Statistical significance is indicated at *p*  < 0.0001^*∗∗∗*^.

In this context, the role of MMP-9 in the animal model of LPS-induced PD was investigated using excised gingival tissue, which was analyzed to determine the expression of mRNA for the metalloproteinase-9. The findings revealed that animals treated with the vehicle exhibited a significantly higher expression of mRNA for MMP-9 compared to those treated with 7HC at doses of 50 and 100 mg/kg, nimesulide at 25 mg/kg, and the naïve group (*p*  < 0.0001). Notably, the results for the 7HC-treated groups were not statistically different to those of the standard drug, suggesting that 7HC possesses bioactive properties that may be beneficial in developing treatments for PD (Figure 4). This finding may elucidate the observed protective effect on bone resorption induced by 7HC, presented in this study.

**Figure 5:**
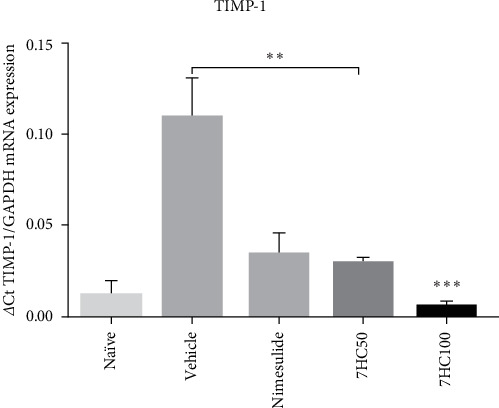
Impact of 7-hydroxycoumarin (7HC) treatment at concentrations of 50 and 100 mg/kg on the mRNA expression of TIMP (tissue inhibitors of metalloproteinases) in gingival tissue excised from C57BL/6 mice with experimental periodontal disease. Data are presented as mean ± standard deviation. Statistical significance is indicated at *p*  < 0.001^*∗∗*^; *p*  < 0.0001^*∗∗∗*^.

An interesting observation was the reduced expression of TIMP-1 (tissue inhibitors of metalloproteinases) in mice treated with 7HC (Figure 5). TIMP is essential for remodeling the extracellular matrix, as it regulates the activity of MMPs [50]. Consequently, it was expected that the treatment would increase in the expression of this protective biomarker. However, the highest levels of TIMP-1 mRNA expression were found in the group of animals receiving the vehicle control, in comparison to those treated with nimesulide at 25 mg/kg, 7HC at both 50 mg/kg (*p*  < 0.001) and 100 mg/kg (*p*  < 0.0001). Given that 7HC inhibits MMP-9, it can be inferred that there was no stimulation of TIMP expression in this scenario.

The study demonstrated that 7HC has significant anti-inflammatory effects by inhibiting the proinflammatory cytokines IL-1β and IL-6. Additionally, it reduces the expression of MMP-9, which is involved in tissue degradation characteristic of periodontitis. At the same time, 7HC increases the expression of mRNA for IL-10 and TGF-β, both of which are cytokines with anti-inflammatory and regenerative roles. This suggests that 7HC may act as an anti-inflammatory bioactive. These findings highlight the potential of 7HC as an adjuvant therapeutic agent in treating PD, aiding in inflammation control and the preservation of periodontal tissues. Its applications in dentistry could range from mouthwashes to targeted delivery systems in periodontal pockets.

It is essential to recognize that 7HC may exhibit toxicity at higher doses. A study involving mice revealed that 7HC is not toxic at doses up to 200 mg/kg [51]. However, research by Gallicchio et al. [52] showed that it inhibits human bone marrow stem cells only at concentrations exceeding 200 μg/mL, suggesting potential toxicity at elevated doses.

In terms of absorption and bioavailability, a Caco-2 cell model demonstrated significant absorption through passive diffusion, with a recovery rate of ~83.31% ± 3.52%. Additionally, Ritschel et al. [53] investigated the bioavailability of umbelliferone in rhesus monkeys at a dose of 1 mg/kg, reporting a half-life of 0.8 ± 0.29 h following intravenous administration, along with a low oral bioavailability of only 17.0% ± 5%.

The study has important limitations, such as the use of an animal model, which complicates the direct extrapolation of results to humans. Furthermore, it lacked assessments of possible systemic side effects and controlled clinical trials. Therefore, additional human studies are essential to validate its safety and clinical efficacy.

## 4. Conclusions

Effectively managing the inflammatory immune response in PD is essential for evaluating treatment outcomes and prognosis. In this study, researchers explored the effects of the compound 7HC in an animal model of LPS-induced PD. The results revealed that 7HC inhibited the expression of proinflammatory cytokines while promoting a shift in the cytokine balance toward a predominance of anti-inflammatory cytokines. Additionally, it reduced the expression of MMP-9, which is linked to the tissue degradation associated with periodontitis. This dual mechanism provided protective effects against alveolar bone resorption. These encouraging findings suggest that 7HC could serve as a valuable bioactive agent for enhancing periodontal therapy, offering hope for improved management of PD.

## Data Availability

The data supporting the findings of this study may be obtained from the corresponding author upon a reasonable request.
